# Acquired radioresistance in cancer associated fibroblasts is concomitant with enhanced antioxidant potential and DNA repair capacity

**DOI:** 10.1186/s12964-021-00711-4

**Published:** 2021-02-26

**Authors:** Jason D. Domogauer, Sonia M. de Toledo, Roger W. Howell, Edouard I. Azzam

**Affiliations:** Division of Radiation Research and Center for Cell Signaling, Department of Radiology, Rutgers Biomedical and Health Sciences, New Jersey Medical School, Rutgers University, 205 South Orange Avenue, Room - F1212, Newark, NJ USA

**Keywords:** Cancer associated fibroblasts, Radiotherapy, Radioresistance, Oxidative metabolism, DNA repair, Intercellular communication

## Abstract

**Background:**

Cancer-associated fibroblasts (CAFs) are a major component of the cancer stroma, and their response to therapeutic treatments likely impacts the outcome. We tested the hypothesis that CAFs develop unique characteristics that enhance their resistance to ionizing radiation.

**Methods:**

CAFs were generated through intimate coculture of normal human fibroblasts of skin or lung origin with various human cancer cell types using permeable microporous membrane inserts. Fibroblasts and cancer cells are grown intimately, yet separately, on either side of the insert’s membrane for extended times to generate activated fibroblast populations highly enriched in CAFs.

**Results:**

The generated CAFs exhibited a decrease in Caveolin-1 protein expression levels, a CAF biomarker, which was further enhanced when the coculture was maintained under in-vivo-like oxygen tension conditions. The level of p21^Waf1^ was also attenuated, a characteristic also associated with accelerated tumor growth. Furthermore, the generated CAFs experienced perturbations in their redox environment as demonstrated by increases in protein carbonylation, mitochondrial superoxide anion levels, and modulation of the activity of the antioxidants, manganese superoxide dismutase and catalase. Propagation of the isolated CAFs for 25 population doublings was associated with enhanced genomic instability and a decrease in expression of the senescence markers β-galactosidase and p16^INK4a^. With relevance to radiotherapeutic treatments, CAFs in coculture with cancer cells of diverse origins (breast, brain, lung, and prostate) were resistant to the clastogenic effects of ^137^Cs γ rays compared to naïve fibroblasts. Addition of repair inhibitors of single- or double-stranded DNA breaks attenuated the resistance of CAFs to the clastogenic effects of γ rays, supporting a role for increased ability to repair DNA damage in CAF radioresistance.

**Conclusions:**

This study reveals that CAFs are radioresistant and experience significant changes in indices of oxidative metabolism. The CAFs that survive radiation treatment likely modulate the fate of the associated cancer cells. Identifying them together with their mode of communication with cancer cells, and eradicating them, particularly when they may exist at the margin of the radiotherapy planning target volume, may improve the efficacy of cancer treatments.
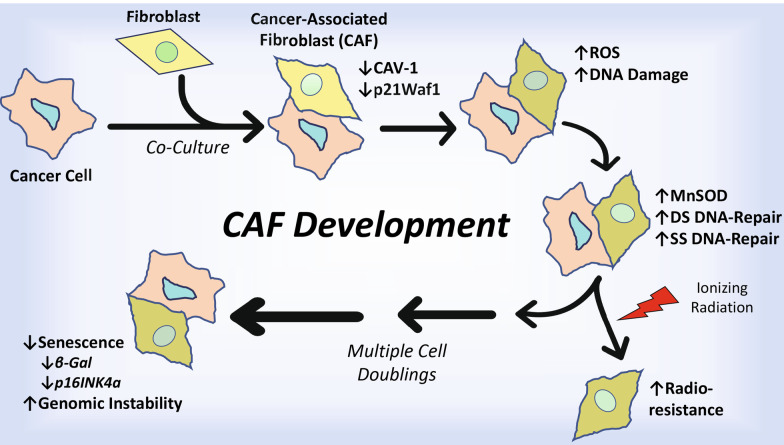

**Video Abstract**

## Background

The tumor microenvironment (TME) is a complex system comprised of parenchymal neoplastic cells that co-exist and evolve alongside a vascularized stroma. This stromal component consists typically of fibroblasts, myofibroblasts, endothelial cells, adipocytes, immune components, as well as an extracellular matrix rich in biologically active molecules [1]. A significant constituent, often much of this stroma, are activated fibroblasts, referred to as cancer-associated fibroblasts or carcinoma-associated fibroblasts (CAFs) interposed between the malignant cells and normal host tissues [2, 3]. In effect, there has been an increasing appreciation that cancer is not merely a disease of the neoplastic cells, but also of the cast of supporting players that together form the malignant tissue. Stromal cells in the tumor are required for nutritional support and for removal of waste products; they contribute to regulating access to fluids and gases, the influx of inflammatory cells, and invasion of neoplastic cells [4]. Therefore, elucidation of the biochemical changes that stromal cells undergo when they are associated with cancer cells is fundamental to understanding the processes implicated in tumor maintenance and the response to therapeutic treatments. It is also relevant for diagnostic interpretations and devising new strategies for enhanced therapeutic interventions, which most often target not only the cancer cells but also other cells in the microenvironment; surviving stromal cells may modulate the fate of the cancer cells, which can occur through various modes of intercellular communication [5].

The CAFs comprise 50–70%, and in some instances up to 90% of a tumor’s volume [6]. Unlike normal, non-activated fibroblasts, they contribute to tumor initiation, progression, angiogenesis, immune-suppression, invasion, metastasis, and recurrence [7, 8] in a wide spectrum of epithelial cell-derived solid cancers, including carcinomas of the breast, prostate, lung, pancreas, skin, colon, esophagus, and ovary [9]. In blood-based cancers (e.g., leukemia, multiple myeloma), stromal cells in the bone marrow, together with blood plasma and newly formed blood vessels, regulate and support the survival of cancer cells [10]. However, the mechanisms underlying CAF development and the exact nature of their contribution to cancer pathogenesis are poorly defined. Understanding the underlying processes is important as clinical evidence has associated a prognostic value to CAFs, correlating their presence to high-grade malignancies, therapy failure, and overall poor prognosis [11]. These clinical observations were supported by in vitro experiments. For example, in coculture, pancreatic carcinoma cells became less sensitive to etoposide when grown together with CAFs [12], and intimate contact between CAFs and melanoma cells reduced the cytotoxic effects of cisplatin [13]. In addition to promoting chemoresistance, CAFs also induce endocrine resistance (reviewed in [14]). Particularly, CAFs play critical roles in resistance to targeted therapeutics such as tamoxifen and Gefitinib [15, 16]. Beyond their contribution to chemo-, endocrine-, and targeted therapy-resistance, CAFs have been implicated in treatment failure and associated poor clinical outcomes following radiotherapy. It has been suggested that CAFs protect associated cancer cells from the lethal effects of ionizing radiation [17–19] as a result of their secreting chemokines, cytokines and growth factors.

Ionizing radiation is an essential regimen in the treatment of many cancers [20]. Its use as a therapeutic modality began shortly after Röntgen’s discovery of X-rays in 1895 [21]. Since, radiation therapy has become one of the most commonly used anticancer therapies [22], with 20–60% of all new cancer cases worldwide being treated with external photon beam radiation as a standard option [23]. Brachytherapy, radiopharmaceutical therapy (e.g., iodine-131, yttrium-90, radium-223), or external particle beam therapy (e.g., energetic protons, carbon ions) are other options. However, our understanding of the relative radiosensitivities of the different cell types that form a tumor remains lacking. If CAFs are found to be radioresistant, this would suggest that those that survive the radiation insults may be able to continue their support of the tumor by various intercellular communication pathways during and after radiotherapy. Emerging evidence indicates that CAFs from non-small cell lung cancer can survive ablative doses of ionizing radiation [24]; however, the mechanisms underlying their radioresistance remains unknown. Here, we have adopted a novel and simple strategy to generate highly enriched CAF populations [25]; we examined key changes acquired during and after their development and focused on their ability to withstand the clastogenic effects of cesium-137 γ rays. To this end, we generated CAFs from two human fibroblast strains (AG1522 and MRC5 derived from skin and lung, respectively) cocultured with various human cancer cell types, predominantly MDA-MB-231 or MCF7 breast cancer cells and examined their ability to withstand oxidative stress and repair radiation-induced DNA damage.

## Methods

### Cells

Apparently normal human diploid fibroblast strains AG01522 D (AG1522) (Coriell Institute) and MRC5 (ATCC, CCL-171), destined to become CAFs, were grown in Eagles’ minimum essential medium (MEM) (Corning CellGro) as described [26]. Breast adenocarcinoma MDA-MB-231 (MDA-MB-231-luc-D3H1, Caliper Life Sciences), glioblastoma multiforme T98G (ATCC, CRL-1690), prostate carcinoma DU 145 (ATCC, HTB-81), and non-small cell lung carcinoma cells NCI-H1299 (ATCC, CRL-5803) are of human origin. They were grown in MEM supplemented with 10% (vol/vol) heat-inactivated fetal bovine serum (FBS), 2 mM l-alanyl-l-glutamine, 10 mg/mL nonessential amino acids, 100 units/mL penicillin, and 100 μg/mL streptomycin. Human breast adenocarcinoma MCF7 cells (ATCC, HTB-22) were grown in Roswell Park Memorial Institute (RPMI) 1640 medium (Corning CellGro) supplemented with 10% (vol/vol) heat-inactivated FBS, 2 mM l-alanyl-l-glutamine, 10 mg/mL nonessential amino acids, and 100 units/mL penicillin and 100 μg/mL streptomycin. Human breast non-tumorigenic epithelial MCF 10A cells (MCF10A) (ATCC, CRL-10318) were grown in Dulbecco’s modified Eagle medium: nutrient mixture F-12 (DMEM/F12) (Corning CellGro) supplemented with 5% (vol/vol) horse serum (Invitrogen), 2 mM l-alanyl-l-glutamine, 2.5 μg/mL Amphotericin b, 0.02 μg/mL EGF, 0.5 μg/mL hydrocortisone, 10 μg/mL insulin, 0.1 μg/mL cholera toxin (Sigma), and 100 units/mL penicillin and 100 μg/mL streptomycin. Prior to coculture with fibroblasts, the tumor cells were incubated in the growth medium used for the fibroblast strains for acclimation purposes. All tumor cells could grow in coculture in the fibroblasts’ growth medium for the duration of the experiments. The cell strains and lines used were mycoplasma free and were authenticated by the supplier or by ATCC authentication service (STR profiling). The AG1522 and MRC5 fibroblasts were used at passage 10–13 and passage 17–20, respectively. The cancer cells were used within ten passages after thawing from liquid nitrogen.

### Coculture

**Fig. 1:**
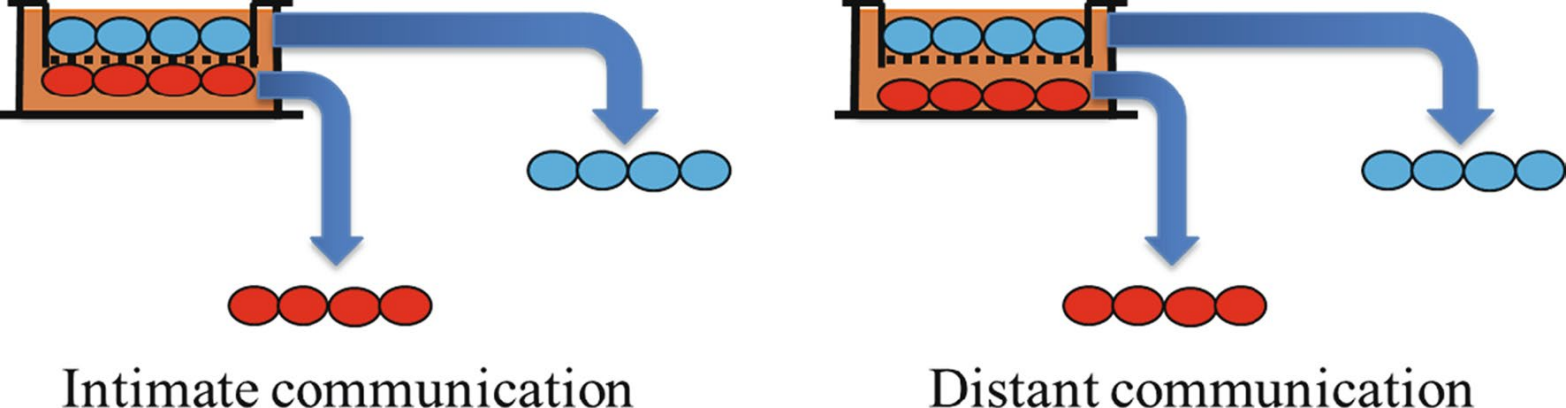
Schematic of coculture strategy with intimate and distant communication for the generation of CAFs. **a** Intimate communication: normal human fibroblasts destined to become CAFs are seeded onto inverted permeable microporous inserts consisting of a 10 μm-thick polyester membrane with 1 μm-pores. Following attachment, the inserts are inverted and placed into the wells of plates and cultured to confluency. Cancer cells or control fibroblasts grown separately in flasks are then seeded on the top side of the inserts with the fibroblasts growing intimately on the bottom side. The cocultured cells are fed every other day and maintained for the desired time. At the respective times following coculture and/or experimental treatments, the CAFs (bottom side of the inserts) and/or cancer cells (top side of the insert) are carefully isolated by trypsinization, yielding highly pure (99.8%) cell populations for analysis or propagation for subsequent studies. **b** Distant communication: Normal human fibroblasts are cultured on the bottom of the well housing the insert, while cancer cells are grown on the top side of the insert

We have examined the effects of both intimate and distant contact between normal fibroblasts and cancer cells for the generation of CAFs. To this end, we employed permeable microporous membrane inserts with 1 μm pores (1.6 × 10^6^ pores/cm^2^; Greiner) immersed in cell culture dishes. The intimate coculture strategy was previously described [25] and is depicted in Fig. 1. Briefly, 2.5 × 10^5^ AG1522 or MRC5 fibroblasts destined to be CAFs were grown on the bottom side of the insert. The fibroblasts were seeded on inverted inserts and allowed to attach, which occurred within 45 min; the inserts were then reinverted and placed in 6-well dishes containing growth medium. The cells were grown for 48 h before they were cocultured with 2.5 × 10^5^ cancer cells seeded on the top side of the insert. After initiation of the coculture, the cells on the insert were fed with fresh medium at 24 and 72 h. The cells were grown in coculture for 120 h, a duration that resulted in a decrease in the levels of Caveolin-1 protein (Cav-1), a known CAF biomarker [27]. Using this coculture model, we were able to generate highly enriched CAF populations (99.8% purity) with in-vivo-like characteristics [25].

For distant communication, AG1522 cells were cultured on the bottom of the well housing the insert, while MDA-MB-231 or MCF7 cells were grown on the top side of the insert. Using this strategy, we were unable to generate CAFs with reduced Cav-1; consequently, the studies were focused on intimate coculture of fibroblasts with cancer cells.

### Analysis of protein expression profile by mass spectrometry (MS)

Mass spectrum measurements of proteins and peptides that are altered in AG1522 cells cocultured intimately with MDA-MB-231 cells were compared with measurements made in AG1522 fibroblast controls. Two cell culture environments were studied: (1) ambient (155 mm Hg; 21% O_2_), and (2) in-vivo-like oxygen tension conditions in breast cancer (7 mm Hg; 0.5% O_2_) environments [28]. The in-vivo-like environment was generated in a G300C Xvivo chamber (BioSpherix™). Cells were acclimated to the humidified hypoxic environment by linearly titrating the O_2_ percentage from ambient to 0.5% over 12 h. The cells were then maintained at 0.5% O_2_ and 5% CO_2_ for the duration of the experiment (120 h), with medium changes occurring at 24 and 72 h post-coculture initiation with growth medium that was also acclimated to the hypoxic environment.

Following growth for 120 h in respective oxygen tension conditions, the cells were removed by Accutase® (Sigma-Aldrich) and washed 3X with PBS. They were suspended in 8 M urea dissolved in 50 mM Tris–HCL containing 1% protease inhibitor cocktail, and ultra-sonicated twice for 10 s. The protein concentration was measured by the Bradford assay. Dithiothreitol (DTT 2 mM, for disulfide bond reduction) and iodoacetamide (4 mM for alkylation) were added to protein lysates followed by digestion with Lys-C and trypsin. The protein digestion solution was desalted by C-18 spin column, and the digested peptides were analyzed by LC–MS/MS on a Q Exactive tandem MS instrument using technical triplicates for each sample. The MS/MS spectral data was searched against Swissprot Human Protein Database. The database search criteria were acetyl (protein N-Term), carbamidomethyl (C), and oxidation (M), 10 ppm for precursor mass tolerance, 0.1 daltons for fragment mass tolerance. The average spectral counts of AG1522 CAFs cocultured with MDA-MB-231 were compared to AG1522 control spectral counts under ambient and hypoxic conditions and only ratio changes greater than 1.5 or less than 0.68 were included in the analysis.

### Western blot analysis

Total cell lysates were prepared in modified radioimmuno-precipitation assay (RIPA) buffer [Tris pH 7.5 50 mM, NaCl 150 mM, NP40 1% (vol/vol), DOC 0.5% (vol/vol), SDS 0.1% (vol/vol)] supplemented with protease and phosphatase inhibitor cocktail (Sigma-Aldrich). Proteins were separated on a 10% (vol/vol) SDS-PAGE gel followed by transfer onto a 0.2 μm nitrocellulose membrane (0.2 μm pore size, Biorad). Antibodies to Cav-1 (1:1000; BD Biosciences), p21^Waf1^ (1:500; Millipore) and p16^INK4a^ (1:300; Santa Cruz) were used in the analyses. Secondary antibodies conjugated with horseradish peroxidase (1:5000; BioRad) and an enhanced chemiluminescence system (PerkinElmer) was used for protein detection. Luminescence was determined by exposure to X-ray film, and densitometry analysis was performed with an Epson scanner and National Institutes of Health Image J software (NIH Research Services Branch, Bethesda, MD). Staining of the nitrocellulose membranes with Ponceau S Red (Sigma-Aldrich) was used to verify equal loading of the samples (loading control) [29].

### In situ immunofluorescence analysis

CAFs growing on the bottom side of the insert were harvested following treatment with Accutase®, rinsed two times in PBS, and suspended in MEM supplemented with 50% (vol/vol) FBS. They were then seeded onto glass coverslips contained within P30 dishes (5 × 10^4^ cells in 250 μL of growth medium). Following attachment, which occurred within 45 min, 2 mL of growth medium was added to the dish. The cells were then incubated in a humidified air atmosphere of 5% (vol/vol) CO_2_ at 37 °C for 48 h. After incubation, the cells were rinsed in PBS and fixed with 4% (wt/vol) paraformaldehyde in PBS for 10 min. After fixation, the cells were rinsed with Tris Buffered Saline (TBS) before being permeabilized with 0.25% (vol/vol) Triton X-100 and 0.1% (wt/vol) saponin in TBS for 5 min. The cells were then blocked with TBS supplemented with 2% (vol/vol) normal goat serum, 1% (wt/vol) bovine serum albumin, and 0.1% (vol/vol) Triton X-100 for 1 h. The blocking buffer was subsequently removed and the cells incubated with anti-Cav-1 (1:1000: BD Biosciences) at 4 °C overnight, followed by incubation with goat-anti-mouse Alexa Fluor® 488-conjugated secondary (1:5000: Molecular Probes) for 1 h. After incubation, the cells were washed at least five times with TBS before being mounted onto glass slides with SlowFade™ Gold antifade mountant with DAPI (Invitrogen). Microscopy was carried out using a 63X oil objective on a Zeiss Axiovert 200M microscope. All images were acquired using identical exposure times and conditions.

### Protein oxidation

Protein carbonyl levels, an index of protein oxidation [30], were determined by immunoblotting using the OxyBlot Oxidized Protein Detection Kit (MilliporeSigma). Briefly, samples containing 20 μg protein extracted from whole cells were derivatized with 2,4-dinitrophenylhydrazine (DNPH) to the corresponding 2,4-dinitrophenylhydrazone (DNP). DNPH-derivatized protein samples were separated by SDS-PAGE, blotted onto nitrocellulose membranes, reacted with anti-DNP antibody (Millipore), and visualized by standard immune techniques.

### MitoSOX measurement

MitoSOX™ Red is a derivative of dihydroethidium, which has selective uptake in actively respiring mitochondria. Once in mitochondria, it is oxidized by superoxide anions producing the red-fluorescent product 2-hydroxyethidium [31]. Cells growing on insert membrane were rinsed with warm Hanks buffered saline solution (HBSS) (Gibco), followed by incubation at 37 °C in the dark for 10 min in HBSS containing 5 μM MitoSOX™ Red (Molecular Probes). CAFs growing on the bottom of the insert were harvested by Accutase®, washed two times with HBSS, and suspended in 400 μL HBSS supplemented with 1% (vol/vol) FBS. Measurements of fluorescence were determined using a FACSCalibur™ flow cytometer (BD Biosciences) with a 488 nm excitation laser and 585/42 nm emission filter. At least 10,000 events were recorded per sample. Relative fluorescent intensity was used as measurement of mitochondrial superoxide production. Results were compared to unstained cells (negative control) and cells treated with medium containing 100 μM Antimycin A, a mitochondrial inhibitor that results in elevated superoxide anion levels (positive control). Analysis was performed using FlowJo V8 software.

### Antioxidant enzyme activity analysis

The activities of superoxide dismutases (SOD) (i.e., MnSOD and CuZnSOD) and catalase were measured by a native in-gel assay [32] as described previously [33]. Fold-change analysis was performed with Image J. The color image was converted to 32-bit gray-scale, followed by a black/white inversion to make the previously achromatic bands black. Intensity of each AG1522 CAF band was measured and compared to its respective control band.

### Senescence

Senescence in AG1522 CAFs was assessed by cytochemical staining for SA-β-galactosidase. AG1522 CAFs were cocultured with MDA-MB-231 or MCF7 breast cancer cells for 120 h. Following coculture, the CAFs were harvested and 7.5 × 10^4^ cells were seeded into 30 mm dishes and cultured for 16 h in a humidified air atmosphere of 5% (vol/vol) CO_2_ at 37 °C. After incubation, the dishes were processed using a Senescence β-Galactosidase Staining Kit (Cell Signaling Technology) at pH 6.0. To quantitate senescence, cells were viewed and counted using a bright-field microscope (40X).

### Irradiation

Following 120 h of coculture, the permeable microporous membrane inserts, containing AG1522 or MRC5 CAFs (bottom side of the insert) and cancer cells (top side of the insert), were exposed to γ rays (effective linear energy transfer (LET) ~ 0.9 keV/μm in liquid water) from a ^137^Cs irradiator (J. L. Shepherd, Mark I, San Fernando, CA). The cultures were placed on a rotating platform to ensure uniform exposure at a mean absorbed dose rate of 50 cGy/min. Control cultures were handled in parallel but were sham treated. Within 15 min after irradiation, the CAFs (bottom side of the insert) and/or cancer cells (top side of the insert) were carefully isolated by trypsinization (yielding 99.8% pure cell populations as assessed by flow cytometry [25], and plated for analyses of micronucleus formation.

### Micronucleus formation

Micronuclei, a form of chromosomal damage that arises mainly from DNA double-strand breaks, are an indicator of radiosensitivity [34–36]. They were evaluated by the cytokinesis block technique [37]. After treatments, the cells were removed from the inserts, and 7.5 × 10^4^ cells were seeded into P30 culture dishes (Cellstar) in the presence of 2 μg/mL (for AG1522) or 1 μg/mL (for MRC5) of cytochalasin B (Sigma, St. Louis, MO) and incubated at 37 °C. After 72 h, the cells were rinsed in normal saline, fixed in ethanol, stained with Hoechst 33342 solution (1 μg/mL dH_2_O), and viewed using a 40X objective on a Leica DM IL fluorescent microscope (Leica Microsystems). At least 500–1000 cells/treatment were examined per group in each experiment, and only micronuclei in binucleated cells were considered for analysis. At the respective concentrations used, cytochalasin B was not toxic to AG1522 or MRC5 fibroblasts.

### Inhibitors of DNA repair

PJ 34 hydrochloride (PJ 34), a poly (ADP-ribose) polymerase (PARP) inhibitor (Tocaris) was added to cell cocultures at a non-toxic concentration of 10 μM at 24 h prior to irradiation. NU 7441, a selective DNA-PK inhibitor (Tocaris) was added to cell cocultures at a non-toxic concentration of 10 μM at 30 min prior to irradiation. The cells were incubated with the various inhibitors until harvest.

### Statistical analysis

Poisson statistics was used to calculate the standard error associated with the percentage of cells with micronuclei over the total number of cells scored. The Pearson’s χ^2^-test was used to compare treatment groups versus respective controls. A *p* value of < 0.05 was considered statistically significant. Unless otherwise indicated, the data shown are representative of at least three independent experiments, and standard errors of the means are indicated in the figures. In each experiment, the CAFs used in analysis of each treatment group originated from 3 to 6 replicate inserts.

## Results

### The effect of intimate contact and distant communication between normal human fibroblasts and breast cancer cells on CAF generation

**Fig. 2:**
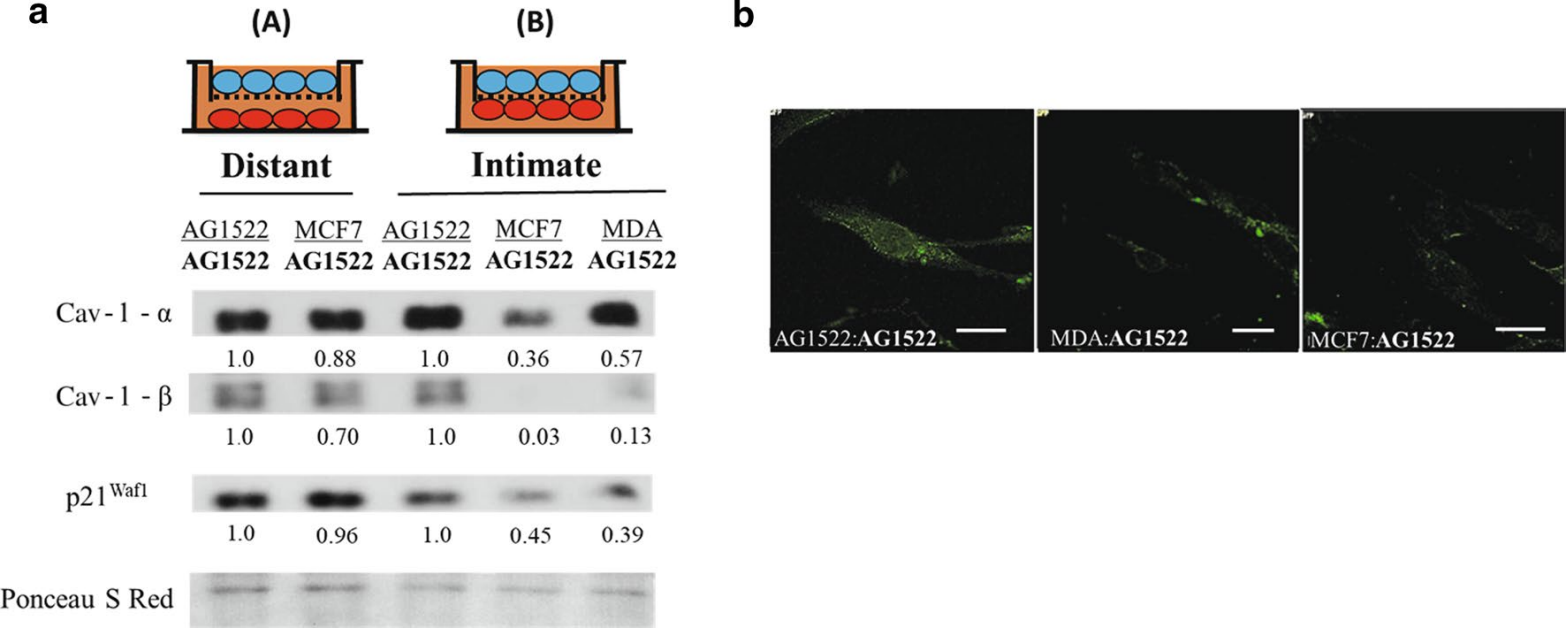
Modulation of caveolin 1 and p21^Waf1^ expression in CAFs generated following intimate or distant coculture of normal human fibroblasts with breast cancer cells. **a** Western blot analyses of Cav-1 and p21^Waf1^ in AG1522 CAFs that were cocultured for 120 h with MCF7, MDA-MB-231, or themselves using the 1 μm permeable microporous membrane inserts. The results indicate that protein expression is altered in the AG1522 CAFs depending upon coculture with MDA-MB-231 or MCF7. Staining with Ponceau S Red was used for loading control and to determine fold-change in expression level. **b** Microscopic images (63X oil objective on a Zeiss Axiovert 200 M) of in situ immunofluorescence (scale bar = 20 μm) of Cav-1 expression in AG1522 cells that had been in co-culture with AG1522 cells (control) (left), or with MDA-MB-231 (center) or MCF7 (right) breast cancer cells for 120 h

Understanding the early heterotypic interactions between cancer cells and the surrounding non-cancer stroma is important for elucidating the events leading to stromal activation and establishment of the TME. To this end, control fibroblasts (intimately cocultured with other AG1522 fibroblasts) and CAF cell lysates from cocultures with MDA-MB-231 cells (maintained under ambient or in vivo-like oxygen tension environment) were collected and analyzed by mass spectrometry for changes in protein expression of CAF markers. Several proteins with documented association to CAF or cancer cells were modulated (Table 1). Relative to control, Laminin b1 (LAMB1) [38] and high mobility group box 1 (HMGB1) [39] were increased similarly in CAFs maintained under both ambient and hypoxic conditions (ratio change was above 1.5 threshold). Signal transducer and activator of transcription 1 (STAT1) [40] and insulin-like growth factor 2 mRNA binding protein 3 (IGF2BP3) [41] were increased under ambient conditions, but decreased under hypoxia, while changes in actin gamma 2 (ACTG2) [42] levels were opposite. The 160-fold increase in ACTG2 in hypoxic CAFs was extremely robust. Notably, a 35% decrease in Cav-1, an established marker of CAFs [43, 44], was observed in CAFs maintained under ambient conditions. An even greater reduction (60%) was observed when the coculture was maintained under hypoxic conditions (Table 1). Immunoblot analyses in AG1522 CAFs cocultured with MDA-MB-231 or MCF7 breast cancer cells under ambient conditions confirmed the decrease in Cav-1 detected by mass spectrometry (Fig. 2a). Within the Western blot, two distinct bands were observed, corresponding to the α and β isoforms of Cav-1 [45]. Both isoforms demonstrate reduced levels in CAFs cocultured with MDA-MB-231 or MCF7, compared to control AG1522 fibroblasts (Fig. 2a). A reduction in the expression level of the cyclin-cyclin dependent kinase inhibitor p21^Waf1^ was also observed. Notably, in-situ immunofluorescence of AG1522 CAFs removed from coculture and grown independently for 48 h on cover slips also demonstrated reduced levels of Cav-1 when compared to controls (Fig. 2b). Therefore, through use of this model, CAF populations can be easily generated from normal diploid human fibroblasts following intimate coculture (120 h) with cancer cells. These CAFs are easily isolated with high purity for subsequent analyses of biological endpoints.

**Table 1:** Modulation of CAF-related proteins in unirradiated AG1522 CAFs cocultured intimately with MDA-MB-231 cells relative to control AG1522 cells

Identified proteins	Function	Fold change (ambient atmosphere)	Fold change (hypoxic at in vivo‐like PO2)
Laminin subunit beta 1 (LAMB1)	Vascular basement membrane structure	3.8	4.0
High mobility group protein B1 (HMGB1)	Chromatin association nuclear protein	3.5	3.2
Caveolin‐1 OS = Homo sapiens (Cav‐1)	Integral membrane protein	0.65	0.36
Signal transducer and activator of transcription 1‐ alpha/beta (STAT1)	Cytoplasmic transcription factor	2.0	0.39
Insulin‐like growth factor 2 mRNA‐binding protein 3 (IGF2BP3)	RNA binding factor	1.7	0.47
Actin, gamma‐enteric smooth muscle (ACTG2)	Cell migration	0.01	160

Examples of proteins with documented association to CAF and/or cancer cells. These proteins were not between the ratio change thresholds of 0.68 and 1.5 and had spectral counts above 2 for all three triplicates from the mass spectra analysis

### CAFs experience altered redox environment and enhanced MnSOD activity

**Fig. 3:**
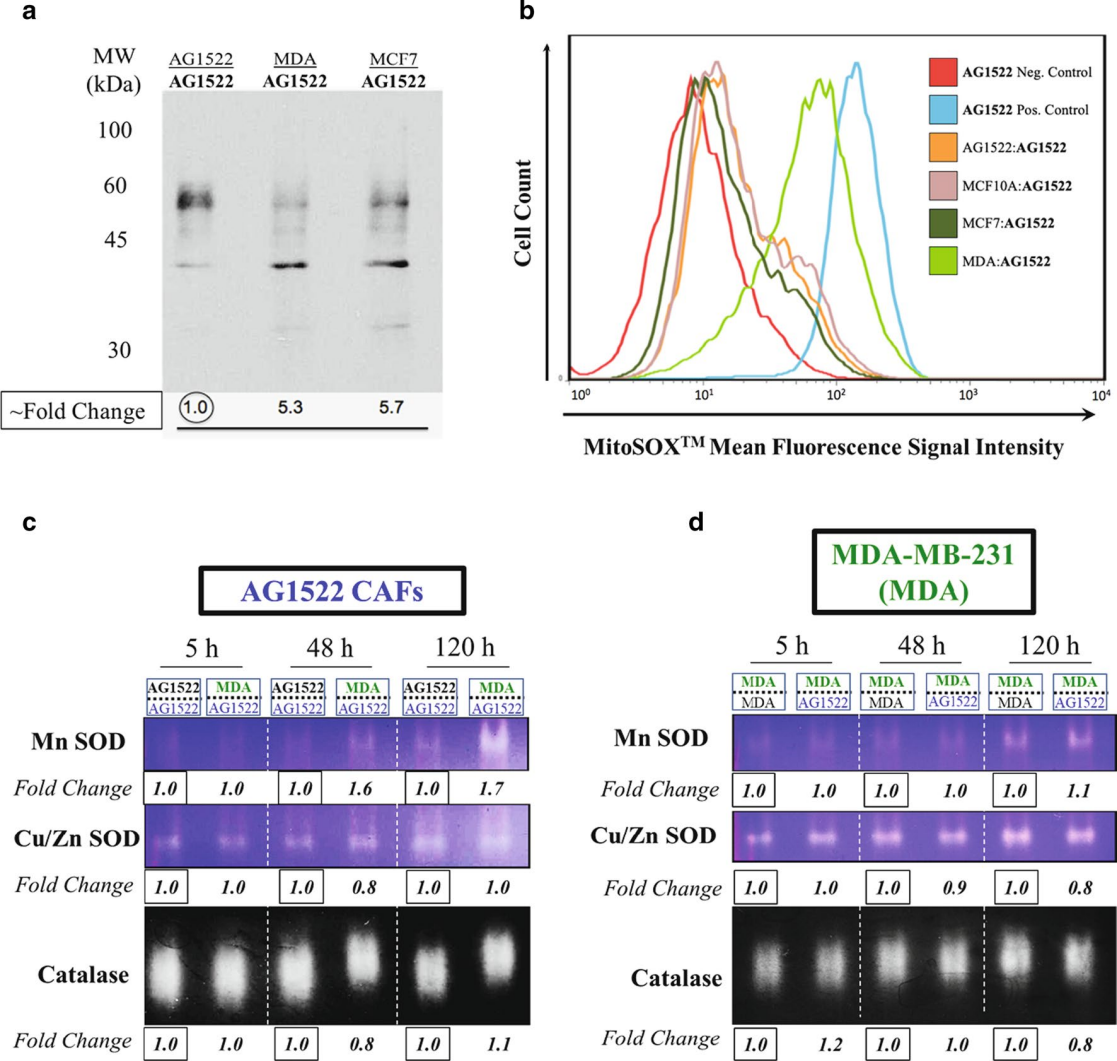
Oxidative stress in AG1522 CAFs that were cocultured with MDA-MB-231 or MCF7 breast cancer cell for 120 h. **a** Protein carbonylation demonstrating alterations in protein oxidation with both increased (~ 40 kDa) and decreased (~ 60 kDa) bands. **b** Induction of mitochondrial oxidative stress: the prevalence of superoxide anion (O_2_^·−^) was analyzed by MitoSOX™ Red and flow cytometry in AG1522 CAFs cocultured with MDA-MB-231 (light green histogram) or MCF7 (dark green histogram) breast cancer cells, and in AG1522 fibroblasts cocultured with MCF10A (purple histogram) non-tumorigenic breast epithelial cells or themselves (orange histogram) (control). Additional controls consisted of AG1522 fibroblasts without MitoSOX™ Red loading (red histogram) (negative control) and AG1522 fibroblasts treated with antimycin A (blue histogram) (positive control). AG1522 CAFs cocultured with MDA-MB-231 (light green) show a ten-fold increase in MitoSox™ levels over negative control (red). The histograms corresponding to AG1522 cocultures with MCF7 (dark green), MCF10A (purple), or AG1522 (orange) possess subpopulations of cells with a higher expression of MitoSOX™ Red (see appearance of bumps in tails of descending parts of the curves compared to the smoother lognormal shape in the negative control) than the larger population; however, no other significant changes were observed for these samples. **c**, **d** Modulation of antioxidant enzyme activity. Antioxidant enzyme activities in **c** AG1522 CAFs and **d** MDA-MB-231 cells cocultured with each other for 5, 48 or 120 h. Coculture with MDA-MB-231 resulted in an increased in MnSOD activity in AG1522 CAFs at 48 and 120 h. Additionally, although the enzyme activity remained unchanged, the catalase band appeared to run slower through the gel in AG1522 CAFs at 48 and 120 h. No changes were noted in CAF CuZnSOD or any of the antioxidant enzymes in the MDA-MB-231 lysate samples. Fold change = relative change. The results are representative of two separate experiments. In each experiment, 5 independent replicates were combined to generate enough cells for analysis

Previous studies have suggested that loss of Cav-1 leads to oxidative stress and mitochondrial impairment in CAFs [46]. To further characterize the CAFs generated in our studies, we analyzed protein oxidation in AG1522 CAFs collected after a 120 h coculture with MDA-MB-231 or MCF7 cells. To this end, we used immunoblotting to detect the formation of protein carbonyl groups (aldehydes and ketones) on protein side chains, which serves to evaluate general oxidative stress within a cell [47]. AG1522 CAFs cocultured with either MDA-MB-231 or MCF7 cells showed similar differences in protein carbonylation, with notable changes including an increase in a band of ~ 40 kDa and a decrease in a band(s) of ~ 60 kDa, when compared to control (Fig. 3a). To gain understanding of the CAFs’ redox environment, we measured the levels of mitochondrial superoxide anions. Mitochondria are the site of cellular respiration and therefore the primary generator of reactive oxygen species (ROS) with the superoxide anion radical (O_2_^·−^) being the major ROS generated [48]. Live CAFs were loaded with MitoSOX™ Red, a fluorogenic dye specifically targeted to mitochondria; the dye fluoresces red when oxidized by superoxide anions (O_2_^·−^), which permits analysis by flow cytometry. AG1522 CAFs cocultured with MDA-MB-231 cells harbored a ten-fold increase in O_2_^·−^ versus AG1522 control, with levels approaching the positive control sample (AG1522 fibroblasts treated with 100 μM of Antimycin A) (Fig. 3b). Alternatively, AG1522 CAFs cocultured with MCF7, and AG1522 fibroblasts cocultured with non-tumorigenic MCF10A, showed no appreciable difference compared to AG1522 control cells (Fig. 3b). To note, histograms corresponding to AG1522 fibroblasts cocultured with MCF7, MCF10A, or AG1522 appear to possess subpopulations of cells with a higher expression of MitoSOX™ Red than the larger population, whereas the histogram corresponding to the AG1522 coculture with MDA-MB-231 has no discernable subpopulation and is more reflective of the lognormal distribution of the control population. Overall, these results show that triple-negative MDA-MB-231 breast cancer cells have a more significant effect on mitochondrial oxidative stress in CAFs.

To ensure protection against excess levels of ROS, cells possess a robust antioxidant defense system comprising glutathione peroxidase, superoxide dismutase (SOD), and catalase among other molecules [49, 50]. To assess whether AG1522 CAFs cocultured with MDA-MB-231 cells had altered antioxidant potential, their endogenous SOD and catalase enzymatic activities were analyzed utilizing a native in-gel assay. Compared to AG1522 controls, activity of the mitochondrion-localized form of SOD (i.e., MnSOD) [51] was increased in CAFs beginning at 48 h and continuing at 120 h (Fig. 3c). No increase in the activity of CuZn SOD, the predominantly cytoplasmic form of SOD [52] was observed (Fig. 3c), supporting the finding that the mitochondria within CAFs are especially susceptible to oxidative stress [53]. Interestingly, while no increase in activity was detected for catalase, the catalase protein band appeared to run slower through the gel in the 48 h and 120 h samples, when compared to controls (Fig. 3c), suggesting post-translation modification of the protein or its complexing with other proteins [54]. Notably, there were no apparent changes in the activity or running patterns of SOD or catalase in MDA-MB-231 cells cocultured with AG1522 CAFs for 5, 48, or 120 h, when compared to MDA-MB-231 controls (Fig. 3d).

### Progeny of isolated CAFs are genomically unstable

**Fig. 4:**
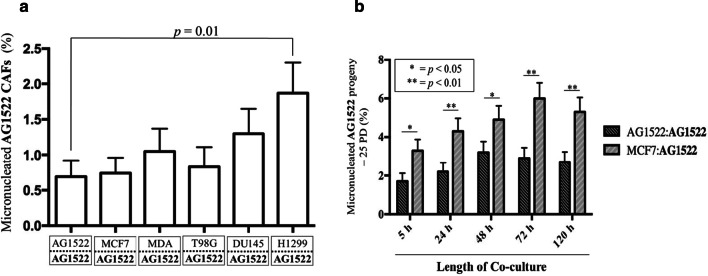
Chromosomal damage in the CAFs and in their progeny. **a** Spontaneous chromosomal damage in CAFs. Micronucleus formation in AG1522 CAFs cocultured with MCF7 or MDA-MB-231 breast cancer cells, T98G glioblastoma cells, DU145 prostate carcinoma cells, or H1299 non-small cell lung cancer cells for 120 h without exposure to ionizing radiation. AG1522 CAFs cocultured with H1299 led to a significant increase in micronuclei levels, when compared to AG1522 control (*p* = 0.01). The results are representative of three separate experiments. **b** Induction of genomic instability in CAF progeny. Micronucleus formation in AG1522 CAF progeny. AG1522 CAFs were cocultured with themselves or MCF7 breast cancer cells for 5, 24, 48, 72, or 120 h, harvested, and subcultured as a single population for 25 population doublings (PDs). AG1522 progeny cocultured with MCF7 for all time periods demonstrated a significant increase in micronuclei frequency (* = *p* < 0.05; ** = *p* < 0.01)

A disruption of the cellular redox environment leading to excess levels of ROS and protein oxidation can lead to degradation of DNA repair proteins, which may result in genomic instability [55]. Interestingly, the genetic integrity of CAFs is an important topic of debate, with some studies reporting a high frequency of genetic aberrations [56], whereas others have reported very low or zero mutation rates [57]. Most of these reports are derived from human tumor samples, potentially complicating the analysis and interpretation of results. In vitro investigations utilizing pure populations of CAFs provide simplified models that permit better insight into the genetic integrity of CAFs. Therefore, AG1522 fibroblasts were cocultured with cancer cells for 120 h, isolated from their respective inserts, and assessed for chromosomal damage in the form of micronuclei. CAFs cocultured with MDA-MB-231, MCF7, T98G, or DU145 cells resulted in no significant difference in micronuclei incidence when compared to AG1522 control; however, a significant difference was observed in CAFs cocultured with H1299 cells (*p* = 0.01) (Fig. 4a), highlighting the heterogeneity among tumors and suggesting that CAFs experience different stresses depending upon the TME in which they are generated. Interestingly, although CAFs cocultured with MDA-MB-231 cells for 120 h experienced high ROS levels (i.e., O_2_^·−^), there appeared to be no significant changes in their genomic integrity at the end of the coculture period as measured by the micronucleus assay, suggesting the elevated MnSOD activity described above may be acting to protect the cells from ROS-induced DNA damage.

Although initial investigations suggested that the majority of CAFs are genetically stable following 120 h of coculture with the majority of the analyzed cancer cell lines, disruption of the cellular redox environment can result in genomic instability that is expressed many cell generations following the primary insult [33, 58]. Such genomic instability can lead to secondary harmful effects including carcinogenesis. Therefore, AG1522 cells were cocultured with MCF7 breast cancer cells for 5, 24, 48, 72, or 120 h in the membrane insert system, harvested, and propagated for 25 population doublings (PDs) in order to assess the genomic integrity of the CAFs’ progeny. An increase in micronucleus frequency was detected in progeny cells derived from parental AG1522 cells cocultured with MCF7 cells for 5 to 120 h (Fig. 4b). The detection of genomic instability in progeny of cells cocultured with cancer cells for only 5 h suggests that in addition to modulation of Cav-1 and other markers and/or perturbations in the redox environment detected at 120 h of coculture, other mechanisms are altered in stromal cells following rather short coculture times with cancer cells and contribute to genomic instability.

### CAFs and senescence

**Fig. 5:**
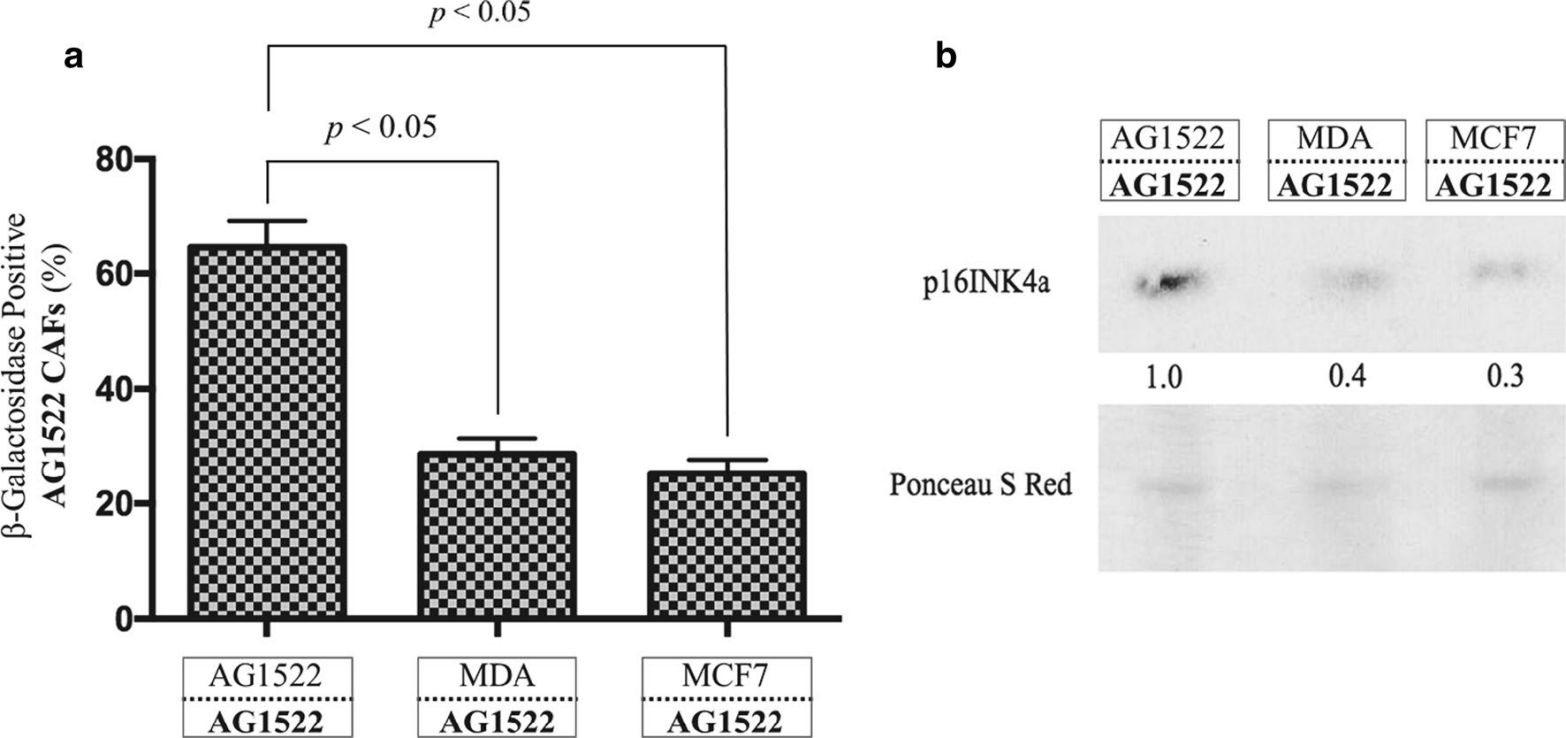
CAFs and senescence. Aged AG1522 CAFs cocultured with MDA-MB-231 or MCF7 breast cancer cells for 120 h have reduced expression of senescence biomarkers. **a** senescence-associated acidic β-galactosidase and **b** p16^INK4a^. The results are representative of three separate experiments

As cancer is typically a disease associated with aging, a concern over CAFs’ progeny may not appear warranted as fibroblasts undergo a limited number of cell divisions before entering an irreversible cell cycle arrest (i.e., senescence) [59]. However, CAFs maintain an increased density because of proliferative activity around tumors [60]. To investigate senescence, we utilized aged AG1522 fibroblasts that have undergone 37 PDs (AG1522 cells senesce at ~ PD 50 [61]) to generate aged CAFs through our coculture protocol. Following 120 h of coculture with cancer cells, the AG1522 CAFs were harvested, allowed to grow for 16 h, and then assessed for expression of senescence-associated acidic β-galactosidase (SA-βGal), a marker of cellular senescence [62]. CAFs cocultured with MDA-MB-231 or MCF7 demonstrated a significant decrease in expression of SA-βGal protein, supporting a decrease in senescence (*p* < 0.05) (Fig. 5a). Senescent cells show striking changes in gene expression, including changes in cell-cycle inhibitors, such as increased levels of the cyclin-dependent kinase inhibitor p16^INK4a^ (reviewed in [63]). Supporting the SA-βGal results, Western blot analysis demonstrated a decrease in p16^INK4a^ in the aged AG1522 CAFs (Fig. 5b).

### CAFs cocultured with breast, prostate, lung, or brain cancer cells acquire radioresistance

**Fig. 6:**
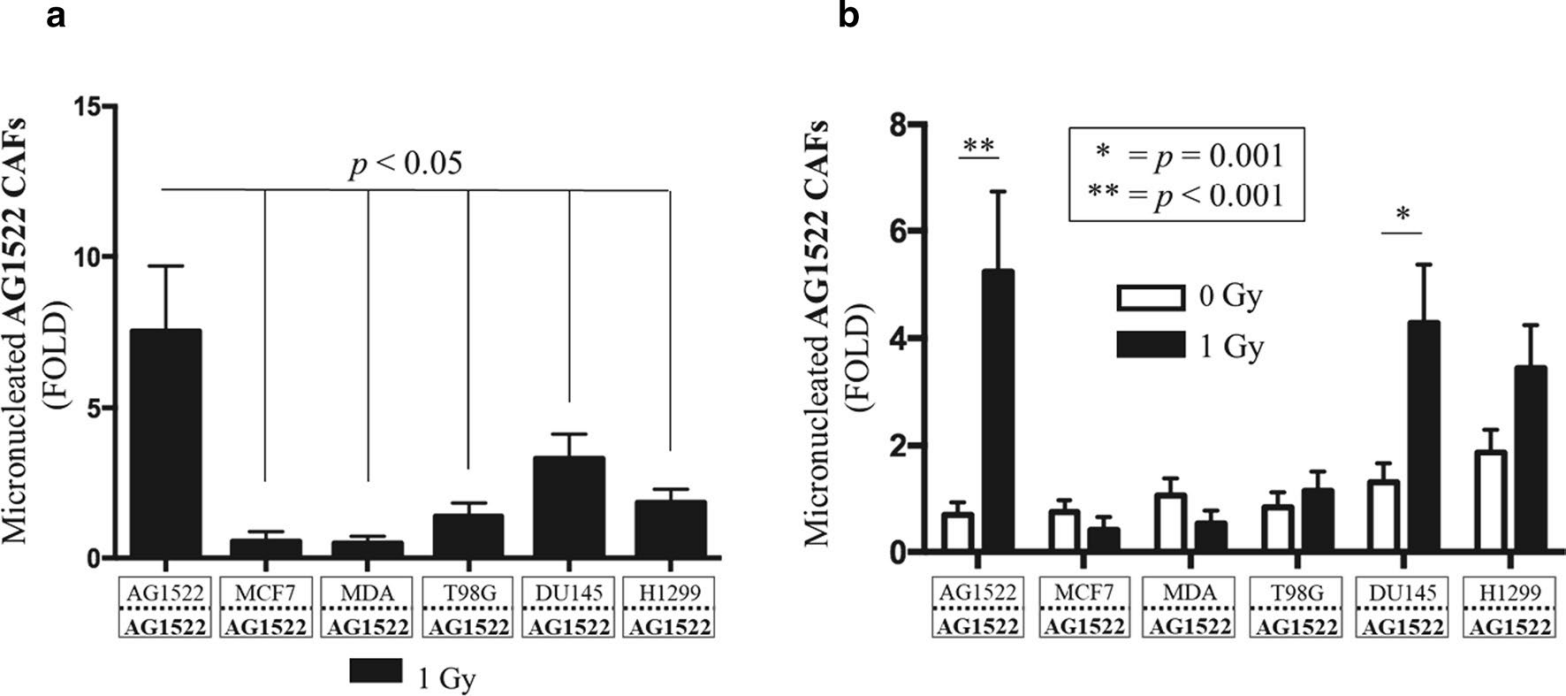
Acquired radioresistance in CAFs cocultured with different cancer cell types. Induction of micronucleus formation in AG1522 CAFs cocultured for 120 h with MCF7 or MDA-MB-231 breast cancer cells, T98G glioblastoma cells, DU145 prostate carcinoma cells, or H1299 non-small cell lung cancer cells, and then irradiated with 1 Gy of ^137^Cs γ rays while in coculture. The fold change in micronuclei are normalized to respective sham-irradiated (0 Gy) controls. CAFs from each coculture showed significantly reduced levels of micronuclei, indicating an increased resistance to ionizing radiation, when compared to AG1522 control (*p* < 0.05). The results are representative of three separate experiments

Micronucleus formation is widely used as an indicator of the cellular sensitivity to radiation [64, 65]. The results in Fig. 6 describe micronucleus formation in AG1522 cells cocultured for 120 h with different cancer cell types and exposed, in coculture, to 1 Gy of ^137^Cs γ rays. Within 15 min after exposure, the AG1522 cells were removed from the insert and assayed for induction of micronuclei. Micronucleus formation in the irradiated samples were normalized to their respective non-irradiated (i.e., sham-irradiated) controls, to generate fold changes in the incidence of micronuclei (henceforth referred to as micronuclei fold changes) as a quantitative measure of CAFs’ response to ionizing radiation. Compared to the irradiated AG1522 fibroblast control cocultured with itself, CAFs irradiated in coculture with MDA-MB-231, MCF7, T98G, DU145, or H1299 cancer cells showed significant reductions in micronuclei fold changes, indicating an enhanced resistance to the DNA damaging effects of γ rays *(p* < 0.05). Taken together, these results indicate that CAFs develop an increased resistance to the clastogenic effects of ionizing radiation regardless of the cancer cell type they are cocultured with. This suggests common events propagated from and/or traits associated with the cancer cells, rather than individual genetic differences, are responsible for the CAF radioresistance.

### CAFs’ acquired radioresistance is cancer cell dependent, not epithelial cell dependent

**Fig. 7:**
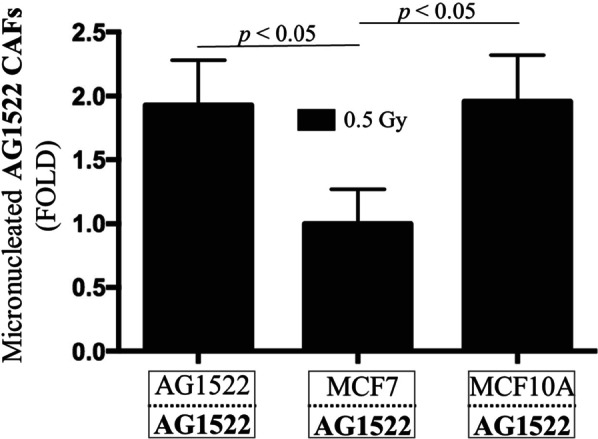
Increased radioresistance of AG1522 CAFs requires coculture with cancer cells versus epithelial cells. Micronucleus formation in AG1522 cells cocultured for 120 h with MCF7 breast cancer cells or MCF10A non-tumorigenic breast epithelial cells, followed by exposure to 0.5 Gy of ^137^Cs γ-rays. Fold changes in micronuclei are normalized to respective sham-irradiated (0 Gy) control. Coculture with MCF10A failed to protect the AG1522 fibroblasts from the radiation insult, whereas coculture with MCF7 significantly reduced the levels of micronuclei in irradiated CAFs. The results are representative of three separate experiments

As fibroblasts and cells of epithelial origin (e.g., carcinoma) are normally separated by the basement membrane, it is important to assess if the observed resistance of CAFs to ionizing radiation is specifically caused by coculture with cancer cells or merely cells of epithelial origin. Therefore, AG1522 fibroblasts were cocultured with non-tumorigenic breast epithelial MCF10A cells for 120 h and exposed to a mean absorbed dose of 0.5 Gy of ^137^Cs γ-rays. Within 15 min after exposure, the AG1522 cells were removed from the insert and assayed for micronuclei formation. Compared to respective control, irradiated AG1522 fibroblasts cocultured with MCF10A did not show a difference in micronuclei fold change. In contrast, a significant difference was observed when AG1522 CAFs were cocultured with MCF7 cancer cells *(p* < 0.05) (Fig. 7). This result supports the premise that cancer cells possess/produce unique factors, independent of their epithelial origin, that are required to generate radioresistant CAFs.

**Fig. 8:**
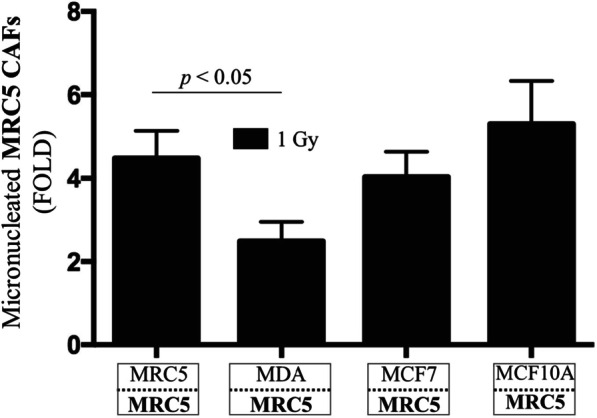
Increased radioresistance of MRC5 CAFs is cancer cell dependent. Micronucleus formation in MRC5 CAFs cocultured for 120 h with MDA-MB-231 or MCF7 breast cancer cells, or MRC5 fibroblasts cocultured with MCF10A non-tumorigenic breast epithelial cells, followed by exposure to 1 Gy of ^137^Cs γ-rays. The cells were γ-irradiated while in coculture. Fold changes in micronuclei are normalized to respective sham-irradiated (0 Gy) control. Only MRC5 CAFs cocultured with MDA-MB-231 demonstrated significantly increased resistance to ionizing radiation, when compared to control (*p* < 0.05). The results are representative of three separate experiments

To test if resistance to ionizing radiation is observed in CAFs derived from different organs, MRC5, a normal human diploid lung fibroblast, was used to create a clinically relevant coculture, as breast cancer commonly metastasizes to the lung [66]. MRC5 fibroblasts were cocultured with MDA-MB-231, MCF7, or MCF10A cells and exposed to a mean absorbed dose 1 Gy of ^137^Cs γ rays. Irradiated samples were normalized to their respective sham-irradiated controls to generate micronuclei fold changes and to assess the CAFs’ response to ionizing radiation. Compared to irradiated MRC5 fibroblasts cocultured with themselves (i.e.*,* control), coculture of the MRC5 CAFs with MDA-MB-231 resulted in significant resistance to ionizing radiation *(p* < 0.05). Interestingly, unlike the AG1522 CAFs, MRC5 CAFs cocultured with MCF7 did not demonstrate significant radioresistance when compared to the MRC5 control (Fig. 8). However, like AG1522 fibroblasts, coculture of MRC5 cells with MCF10A also did not result in significant enhancement in radioresistance (Fig. 8). The absence of radioresistance in MRC5 CAFs cocultured with MCF7 suggests that the development of CAF radioresistance is dependent on the CAF tissue of origin and/or the specific properties of the cancer cell subtype. Lastly, there was no significant difference in micronuclei levels in sham-irradiated MRC5 cocultured with MDA-MB-231, MCF7, or MCF10A when compared to sham-irradiated AG1522 cells cocultured with itself, demonstrating that the 120 h coculture alone does not result in spontaneous DNA damage in the MRC5 fibroblasts (data not shown).

### Acquired radioresistance of AG1522 and MRC5 CAFs involves enhanced DNA single-strand and double-strand break repair mechanisms

In addition to enhanced antioxidant potential, an increased ability to repair DNA damage has also been correlated with increased resistance to ionizing radiation [67, 68]. Therefore, the roles of DNA single-strand break (SSB) and double-strand break (DSB) repair mechanisms were investigated to determine whether they contribute to CAF radioresistance. AG1522 and MRC5 CAFs were generated through coculture with MDA-MB-231 cells and cultured in the presence of DNA SSB (PJ 34) and DSB (NU 7441) repair inhibitors for 24 h or 0.5 h, respectively. The inserts were then exposed to a mean absorbed dose 1 Gy of ^137^Cs γ-rays in the presence of the inhibitors and the CAFs were analyzed for the formation of micronuclei.

**Fig. 9:**
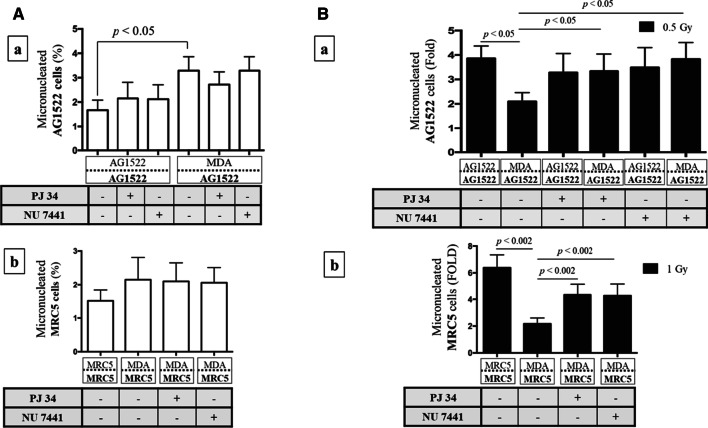
Involvement of DNA repair in CAF radioresistance. **A** Effect of DNA repair inhibitors on basal levels of DNA damage: Micronucleus formation in (a) AG1522 or (b) MRC5 cells cocultured for 120 h with themselves or MDA-MB-231 cells, followed by the addition of DNA SSB (PJ 34) or DSB (NU 7441) repair inhibitors. The inhibitors did not significantly increase basal micronuclei frequency. There was a significant increase in micronuclei frequency in AG1522 CAFs following coculture with MDA-MB-231 (*p* < 0.05). The results are representative of three separate experiments. **B** Radioresistance of CAFs and its mediation by DNA repair: Micronucleus formation in (a) AG1522 or (b) MRC5 CAFs cocultured for 120 h with MDA-MB-231, followed by the addition of DNA SSB (PJ 34) or DSB (NU 7441) repair inhibitors and exposure while in coculture to 0.5 Gy or 1 Gy of ^137^Cs γ-rays, respectively. Values in fold-change are normalized to respective sham-irradiated control. The addition of either inhibitor attenuated the protection against the radiation insult in both the AG1522 and MRC5 CAFs. The results are representative of three separate experiments

Coculture of AG1522 or MRC5 fibroblasts with themselves or with MDA-MB-231 cells in presence of the DNA repair inhibitors, without radiation exposure, did not significantly affect their basal micronuclei levels (Fig. 9A, sub-panels a and b, respectively), consistent with previous observations [68]. However, exposure of the coculture to ^137^Cs γ rays in the presence of the inhibitors led to a significant increase in micronuclei in AG1522 and MRC5 CAFs cocultured with MDA-MB-231 versus respective control without the inhibitors (*p* < 0.05) (Fig. 9B, sub-panels c and d, respectively). Notably, micronucleus formation in AG1522 and MRC5 CAFs cocultured with MDA-MB-231 in the absence of inhibitors confirmed the results in Fig. 6 showing a significant resistance to ionizing radiation (*p* < 0.05). Therefore, an increased DNA repair capacity has a significant role in the radioresistance of CAFs.

## Discussion

Over the last four decades, the interaction between the extracellular matrix (ECM) and tumor cells [69], and between fibroblasts (i.e., CAFs) and tumor cells [70], has been under intense investigation. Initial studies showed that non-cancerous components of the TME could act as tumor-suppressors or tumor-promoters, depending upon diverse variables such as tumor type, stage of disease, stromal components, or the status of certain receptors on the cell surface [71]. These early observations contributed to the current understanding that the TME is capable of regulating gene expression in both cancer cells and components of the non-cancer stroma [72], which shifted the view of the ‘soil’ from a passive player to one of an active director and expanded our understanding of the diversity in cancer phenotypes [73]. The consensus is that the study of malignancy can no longer solely focus on the cancer cells, rather a better understanding of the interactions between cancer cells and their non-cancer stromal components is required. Clearly, this is a challenging task due the intricate web of intercellular crosstalks between the different cellular components, tumor heterogeneity, and the complexity of signaling cascades operating within the TME. Here we used a simple procedure to generate an enriched population of CAFs; we characterized key events associated with their development and their responses to ionizing radiation, a modality used extensively in cancer therapy. An understanding of the CAFs’ response to ionizing radiation may significantly impact treatment planning.

We showed that CAFs can be easily generated by co-culturing apparently normal human diploid fibroblasts with various cancer cell types, primarily breast adenocarcinoma cells, utilizing a permeable microporous-membrane-insert tumor-microenvironment model (Fig. 1). By using this model, CAFs are isolated from the coculture with a high degree of purity (99.8%) [25]. Collectively, our results demonstrate that CAFs generally possess unique characteristics distinct from their fibroblast origin. The isolated CAFs were verified by a consistent decrease in Cav-1 protein levels, with an even greater reduction detected under in-vivo-like pO_2_, which is below ambient (Table 1, Fig. 2). The CAFs also experienced a decrease in expression of p21^Waf1^, which when decreased in stromal fibroblasts results in accelerated growth of MCF7 or MDA-MB-231 tumor xenografts [74]. Additionally, we provide further evidence that CAF generation requires intimate communication with cancer cells. In our tissue culture model, we could not generate CAFs when cancer cells were in distant communication with the fibroblasts (Figs. 1, 2).

We found that CAFs of skin origin (AG1522) and lung origin (MRC5) were more resistant to ionizing radiation compared to their normal fibroblast counterparts. Specifically, AG1522 CAFs cocultured with different cancer cell types (breast, prostate, lung, brain) developed increased resistance to the clastogenic effects of ^137^Cs γ rays (Fig. 6). This finding suggests that traits associated with the cancer phenotype rather than individual genetic differences contribute to radioresistance of the AG1522 CAFs. Furthermore, CAFs’ radioresistance developed following coculture with cancer cells and not with non-malignant epithelial cells (Fig. 7). Significantly, radioresistance of CAFs was associated with a greater capacity for repair of single- and double-strand DNA breaks (Fig. 9). The radioresistance greatly depended upon the tissue of origin from which the CAFs originated (Fig. 8): whereas AG1522 CAFs developed general radioresistance regardless of the cancer type, MRC5 CAFs were radioresistant when cocultured with triple negative MDA-MB-231 cells, but not hormone-dependent MCF7 cells. Omic studies in MRC5 CAFs cocultured with MDA-MB-231 or MCF7 cells may shed light on the factors underlying their differential responses to radiation (Fig. 8). Expansion of these studies to fibroblasts cocultured with cancer cells derived from the same organ/tissue would further inform on the role of the tumor microenvironment in which both cell types reside and have evolved. Metabolic, epigenetic, and other changes induced in CAFs as a result of cues originating from the tumor microenvironment may affect their response to ionizing radiation.

We show that the progeny of CAFs not exposed to radiation are genomically unstable (Fig. 4b). The progeny cells also express reduced levels of senescence markers (β-galacosidase, p16^INK4a^; Fig. 5). Therefore, long-lived CAFs and those that survive radiation treatment may communicate stress factors to other cells, hence amplifying secondary adverse effects [75]. Our emerging data (not shown) indicate that secreted factors as well as the permeability properties of connexin junctional channels linking malignant cells and CAFs together modulate these effects. Thorough characterization of the effects of secreted factors, or those directly communicated between CAFs and cancer cells, on the radiation sensitivity of either the CAFs or cancer cells may provide insight for refining adjuvant therapies. It is also of interest to examine senescence markers and telomere dysfunction in progeny of CAFs surviving irradiation. Notably, senescence escape has been shown to be tightly associated with tumor progression [76].

A decrease in Cav-1 was shown to be associated with increased oxidative stress [46], and perturbations in oxidative metabolism is a hallmark of carcinogenesis that greatly affects short- and long-term effects of ionizing radiation [49]. Therefore, we examined oxidative stress markers in our generated CAFs. We identified a significant increase in oxidative stress in CAFs, as measured by protein carbonylation and elevated levels of mitochondrial superoxide anion (Fig. 3). Interestingly, this stress was associated with an increase in MnSOD antioxidant activity, thereby supporting an attempt of the CAFs to regulate their redox environment. This finding is noteworthy when one considers the phenomenon of the adaptive response by which an increase in antioxidant activity elicited by a small priming dose provides protection against a challenge dose of ionizing radiation or other oxidizing agents, including chemotherapies [50, 77]. Notably, previous findings have shown that elevated antioxidant enzyme activities, especially MnSOD, contribute to radioresistance [78]. Nevertheless, the mechanism by which reduced Cav-1 expression in CAFs leads to an altered oxidative environment, and its potential impact on the response to γ rays, requires further study. Collectively, these unique characteristics of CAFs allow them to act as key modulators of cancer and non-cancer cells within the TME.

## Conclusion

As the burden of cancer continues to grow worldwide, cancer research and treatments can no longer focus solely on the cancer cells but need to be expanded to address the role(s) of the TME. The accumulating evidence is that the TME is complex and consists of many elements that induce physiological changes relevant to cancer progression. Among the many constituents of the TME, CAFs may contribute significantly to cancer pathogenesis and response(s) to therapy. Identifying CAFs and eradicating them during treatment, particularly when they may exist at the margin of the radiotherapy PTV, could improve efficacy of the treatments [79, 80].

Future work examining the fidelity of DNA repair (error free versus error prone) and the key signaling events underlying the enhanced ability of CAFs to repair radiation-induced DNA damage will increase our understanding of how the TME evolves during and after radiotherapy. The knowledge gained is of benefit to developing new and more effective treatment protocols. Although this study has identified the decrease in Cav-1 levels to be a general trait associated with CAF development, understanding the biological processes that regulate this decrease will improve our understanding of a major constituent of the TME. Our proteomic study suggests that a broad spectrum of molecular drivers contribute to CAFs’ development. Unraveling the interaction of these drivers (genetic and epigentic) with each other will likely lead to discovery of additional key events associated with adverse outcome pathways that contribute to the development and strengthening of the TME that supports tumor progression [75]. By unveiling novel mechanisms, our newfound understanding of basic concepts associated with neoplasia will help discover new therapeutic targets and interventions. Whereas we found CAFs to harbor a high level of oxidative stress, examination of the contributing factors (e.g., respiratory rate) and additional protective mechanisms (e.g., mitophagy) should enhance our understanding of how CAFs adapt to oxidative stress, facilitate redox regulators to resist being eliminated by radiation therapy insults, as well as the cause of genomic alterations in their progeny. Additional work is needed to dissect the role of cytokines and other secreted factors in recruitment of cells to the TME, and how these are modulated in response to radiation.

In our studies, CAFs’ radioresistance was assessed via measurements of micronucleus formation, a sensitive marker of radiation sensitivity. Whereas assessments of residual DNA damage following irradiation highly correlates with clonogenic survival measurements [34, 36, 81, 82], evaluating both of these indicators in CAFs exposed to a broad range of γ ray absorbed doses (1–10 Gy) when in coculture with cancer cells would inform on responses to doses similar to those that patients receive during radiotherapy and would be relevant to predictive assays [83, 84]. Finally, investigation of CAFs’ response to hypo- and hyper-fractionation of external photon beams may contribute to our understanding of cell and tissue responses to regimens used clinically [85]. Such research may aid in determining which fractionation protocol would provide the most benefit, based upon the CAF composition of the TME. Furthermore, efforts aimed at identifying CAFs’ response to other modalities of radiotherapy (e.g., proton and carbon ion beam therapies) could provide important insight into the underlying mechanisms of CAFs’ radioresistance. Perhaps most exciting is the development of theranostic approaches that seek to image and eliminate CAFs with radiopharmaceuticals [86]. Such radiopharmaceutical approaches also have the capacity to leverage radiation-induced bystander effects that may ultimately play an important role in eliminating both the CAFs and the cancer cells they support [87, 88]. This work together with long-term follow-up of patients treated with various modes of radiotherapy may provide an opportunity to develop more effective cancer therapies utilizing radiation. It may improve our understanding of collateral adverse side-effects that these therapies may have on accelerating degenerative conditions as well as the emergence of new pathologies [5]. The direct and indirect intercellular communications that CAFs have with their matrix and with normal cells in their vicinity may facilitate the development of these long-term effects. Experimental approaches using antagonists/inhibitors to the effects of candidate molecules propagated from CAFs that survive therapeutic treatments will shed light on ways to manipulate bystander responses towards improved therapeutic outcomes.

## Data Availability

Original results will be supplied upon request.

## Abbreviations

α-SMA: α-Smooth muscle actin
CAF: Cancer-associated fibroblast
Cav-1: Caveolin-1
CuZnSOD: Copper-zinc superoxide dismutase
DAPI: 4′,6-Diamidino-2-phenylindole
DMEM/F12: Dulbecco’s modified Eagle medium: nutrient mixture F-12
DNP: 2,4-Dinitrophenylhydrazone
DNPH: 2,4-Dinitrophenylhydrazine
DOC: Sodium deoxycholate monohydrate
DSB: Double-strand break
DTT: Dithiothreitol
ECM: Extracellular matrix
EGF: Epidermal growth factor
FBS: Fetal bovine serum
Gy: Gray (unit of absorbed dose; equal to one joule of radiation energy absorbed per kilogram of tissue)
h: Hour
HBSS: Hanks’ balanced salt solution
HMGB1: High-mobility group protein B1
LET: Linear energy transfer
LC: Liquid chromatography
MEM: Eagle’s minimum essential medium
MnSOD: Manganese superoxide dismutase
MS: Mass spectrometry
MS/MS: Tandem mass spectrometry
PARP: Poly (ADP-ribose) polymerase
PBS: Phosphate-buffered saline
PD: Population doubling
PJ 34: PJ 34 hydrochloride
PTV: Planning target volume
RIPA: Radioimmunoprecipitation assay
ROS: Reactive oxygen species
RPMI: Roswell Park Memorial Institute 1640
SA-βGal: Senescence-associated acidic β-galactosidase
SDS: Sodium dodecyl sulfate
SDS-PAGE: Sodium dodecyl sulfate polyacrylamide gel electrophoresis
SOD: Superoxide dismutase
SSB: Single-strand break
TBS: Tris-buffered saline
TME: Tumor microenvironment
